# A novel method for credit scoring based on feature transformation and ensemble model

**DOI:** 10.7717/peerj-cs.579

**Published:** 2021-06-04

**Authors:** Hongxiang Li, Ao Feng, Bin Lin, Houcheng Su, Zixi Liu, Xuliang Duan, Haibo Pu, Yifei Wang

**Affiliations:** 1 College of Information Engineering, Sichuan Agricultural University, Ya’an, Sichuan, China; 2 Research Institute of Economics and Management, Southwest University of Finance & Economics, Chengdu, Sichuan, China

**Keywords:** Boosting tree, AutoEncoder, Feature transformation, Credit scoring, Deep neural network, Factorization machine

## Abstract

Credit scoring is a very critical task for banks and other financial institutions, and it has become an important evaluation metric to distinguish potential defaulting users. In this paper, we propose a credit score prediction method based on feature transformation and ensemble model, which is essentially a cascade approach. The feature transformation process consisting of boosting trees (BT) and auto-encoders (AE) is employed to replace manual feature engineering and to solve the data imbalance problem. For the classification process, this paper designs a heterogeneous ensemble model by weighting the factorization machine (FM) and deep neural networks (DNN), which can efficiently extract low-order intersections and high-order intersections. Comprehensive experiments were conducted on two standard datasets and the results demonstrate that the proposed approach outperforms existing credit scoring models in accuracy.

## Introduction

Credit card and loan business is the main way of profit for banks and other financial institutions; however, these two transactions also exist high risks. Improper credit risk control strategy will lead to huge financial losses for financial institutions. According to the data of the People’s Bank of China (http://data.eastmoney.com/cjsj/xzxd.html), as of January 2019, the credit center of the People’s Bank of China has registered a total of 990 million natural persons, of which the number of people with credit records reached 530 million. There was a total of 20,030.9 billion yuan of cumulative new credit in 2020 and 2019. The total new credit will be 1,688,341 billion yuan in 2020 and 1,567,241 billion yuan in 2018. According to data released by the China Association of Banks, the outstanding credit balance of China’s bank card industry totaled 7.59 trillion yuan at the end of 2019, an increase of 10.8% over the previous year.

The subprime mortgage crisis during 2007–2010 was due to the inefficient and low accurate credit scoring methods (Bhatore et al., 2020). In order to reduce potential non-performing assets and improve the efficiency of credit risk control, more reliable credit scoring approaches are urgently demanded. Since it is difficult for financial institutions to decide whether can extend loans to customers, therefore, it is a major task for financial institutions to build high-risk customer identification models and thus control credit risk by using user credit data, behavioral data, and other information. Regulators, for example the European Central Bank, recommend to employ the features extracted from the structured and unstructured data for early warning of credit risk (Lang, Peltonen & Sarlin, 2018). Currently, many fintech companies use customer information to provide credit scoring services for unsecured lending platforms (Jagtiani & Lemieux, 2019).

In order to discover some relationships between the user’s data characteristics and credit assessment, the traditional approach of credit risk assessment is to apply some sort of classification technique to the user’s historic data, such as consumption history, income status, loan status, etc., (Li, Li & Li, 2019b). Many statistical models and optimization techniques, such as linear discriminant analysis, logistic regression, K-nearest neighbor algorithm, maximum likelihood estimation, and conditional random field have been widely applied to credit risk assessment and modeling tasks. Although these techniques can be applied to credit risk assessment, they can be further improved. In the last two decades, there has been a growing approaches proposed in the field of machine learning that can handle large amounts of data yet guarantee good accuracy (Bhatore et al., 2020). For example, machine learning techniques such as Bayesian networks, decision trees, and support vector machines have been widely applied to user credit assessment. Masmoudi, Abid & Masmoudi (2019) used a discrete Bayesian network containing potential vectors for user payment default prediction. Caruso et al. (2020) focused on the correlation relationship between the quantitative and qualitative features of applicants and proposed a hybrid data-based clustering analysis technique for credit risk assessment. Li et al. (2019a) used the concept of migration learning for automated credit assessment, migrating data from traditional business to new business and building predictive models.

Although there are many techniques already available for credit assessment, all the above methods still exist certain limitations. The following challenges remain in credit assessment.

(1) Data imbalance. Since the number of users with high credit risk is less than the common users in real credit assessment tasks, which makes it difficult for traditional methods to achieve satisfactory performance.

(2) Manual feature engineering. To accurately discover the relationship between credit assessment and user characteristics, the manual feature engineering method is usually used. However, it is extremely difficult for financial industry practitioners that has no data science background.

(3) Unsatisfied accuracy. The current performance of data analysis is unsatisfied. Both missed and false positives decisions can cause losses to the banking and financial industry. The low accuracy can be attributed to improper feature selection, inapplicable feature engineering, inefficient parameter tuning, and data imbalance (Hakak et al., 2021).

To address the above challenges, in this paper, we propose a new approach based on feature transformation and ensemble model for credit scoring. To reduce manual feature engineering, we use boosted trees for feature transformation while employ the automatic feature engineering to obtain higher-order representations of the original features in the tree model. Majority class samples are used to train the autoencoder, which changes the feature representation and feature distribution of low and high credit risk customers by calculating reconstruction error features, enabling the prediction model to identify high credit risk customers well in the presence of data imbalance. We construct a prediction model by fusing a factorization machine and a deep neural network, which enables the model to better mine the information among sample features and improve prediction accuracy and robustness.

The main contributions of this paper are as follows.

(1) A trainable automatic feature engineering module is proposed, which does not rely on any manual feature engineering and requires only raw features for feature extraction, feature filtering and feature combination.

(2) A trainable module for solving data imbalance is proposed, and it is also part of automatic feature engineering, which effectively improves the performance of the model in the case of data imbalance.

(3) An approach based on feature transformation and integrated model is proposed for credit scoring, where predictive models are constructed by fusing factor decomposers and deep neural networks in the classification phase.

(4) Experiments are conducted on two benchmark datasets and the results demonstrate that the proposed method significantly outperforms other existing methods. It can help banks and financial institutions to perform efficient and accurate user credit scoring and identify high-risk users from a large number of users.

This paper is organized as follows. In ‘Related Work’, we review the related literature. In ‘Methods and Materials’, the algorithm and model used in this paper are described, and the structure of the proposed hybrid model and the use of reconstruction error to solve the data imbalance problem are presented. In ‘Results’, the experimental part is discussed, including the presentation of the dataset and the comparison of the model performance. In ‘Discussion’, the implications of the proposed approach for credit scoring are discussed. In ‘Conclusion’, a summary of the paper and the directions for future work are provided.

## Related Work

With the development of computer technology, machine learning and deep learning have been widely used in lots of areas, such as image recognition (Oyewola et al., 2021), natural language processing (Sitaula, Basnet & Aryal, 2021), anomaly detection (Hakak et al., 2020) and robotics (Khairuddin et al., 2021), and are becoming mainstream solutions. Many researchers continue to explore various machine learning and deep learning techniques to improve the accuracy of credit risk assessment (Li et al., 2018), as shown in Table 1. Kulkarni, Dhage & Systems (2019) use social media information and machine learning to score customers’ credit. Moula, Guotai & Abedin (2017) conducted comparative experiments on six different databases in the credit prediction domain and the experimental results showed that the robustness and accuracy of support vector machine (SVM) models outperformed classification and regression trees (CART). Bakoben, Bellotti & Adams (2020) used clustering analysis to evaluate credit risk, which shows that unsupervised learning can also achieve favorable performance. Zhang et al. (2017) proposed a flexible neural tree (FNT) based credit risk assessment method for loan applicants. Fan & Yang (2018) proposed a neural network working model based on denoising autoencoder for overcoming data noise.

**Table 1:** Summary of related work.

References	Methods and materials
Moula, Guotai & Abedin (2017)	Support vector machine, classification and regression trees
Zhang et al. (2017)	Flexible neural tree
AghaeiRad, Chen & Ribeiro (2017)	Self-organizing map, feedforward neural network
Dahiya, Handa & Singh (2017)	Hybrid bagging algorithm, feature selection
Fan & Yang (2018)	Denoising autoencoder
Xia et al. (2018)	Heterogeneous integration model, bagging, stacking
Jadhav, He & Jenkins (2018)	Information gain, GA Wrapper
Kulkarni, Dhage & Systems (2019)	Media information, machine learning
Ebenuwa et al. (2019)	Variance ranking technique, ranked order similarity
Pes (2019)	Ensemble models, feature engineering
Bakoben, Bellotti & Adams (2020)	Clustering analysis
Reddy et al. (2020)	Ensemble model
Wang et al. (2020)	Local distribution-based adaptive minority oversampling
Arora & Kaur (2020)	Bootstrap-lasso
Swarna et al. (2020)	Hybrid PCA-GWO

Ensemble or hybrid models enable multiple models to complement each other to form a strong learner, which often performs better than a single model. In recent years, many researchers have proposed credit scoring models that are heterogeneously ensemble or hybrid trained. AghaeiRad, Chen & Ribeiro (2017) proposed a hybrid approach of self-organizing map (SOM) and feedforward neural network (FNN), illustrating that combining supervised and unsupervised learning can effectively improve classification accuracy. Xia et al. (2017) proposed a new heterogeneous integration model combining bagging and stacking and significantly outperformed several state-of-the-art benchmark models. Dahiya, Handa & Singh (2017) used a hybrid bagging algorithm based on feature selection to improve credit risk evaluation, reducing computational complexity while improving model performance. Reddy et al. (2020) proposed an ensemble model consisting of random forest classifier, decision tree classifier, Adaboost classifier, K-nearest neighbor classifier, and logistic regression classifier to improve the performance of existing machine learning methods. Hakak et al. (2021) constructed an ensemble model by extracting salient features and ensemble models to achieve optimization of accuracy and training time.

In realistic credit default forecasting, the number of samples in different categories varies significantly. Typically, the number of high credit risk customers is much smaller than the number of low credit risk customers. In traditional machine learning research, most classification algorithms assume that the prior probabilities of each class of samples are uniformly distributed and the cost of the classifier to misclassify each class of samples is the same. In the case of data imbalance, the information of the majority class samples will overwhelm the information of the minority class samples, making the classifier overly focus on the majority class samples (Hothorn, 2020). However, in credit default prediction, accurately identifying a small number of high credit risk customers has greater value than accurately identifying most low credit risk customers, and banks and financial institutions prefer to improve the classification accuracy of high credit risk customers with small sample sizes. Researchers usually use undersampling and oversampling techniques to change the original distribution of the data by reducing the number of majority class samples and increasing the number of minority class samples in the training set. Wang et al. (2020) proposed a local distribution-based adaptive minority oversampling (LAMO) to deal with the imbalance problem. Venkatraman, Alazab & Networks (2018) proposed a hybrid method of feature-based and image-based similarity mining visualization for label-free anomaly detection. Ebenuwa et al. (2019) proposed a variance ranking technique and ranked order similarity (ROS) when data imbalance, and this method provides an effective technical tool when data imbalance and other similarity measurement techniques are not applicable.

Data and features determine the upper limit of machine learning performance, and the improvements of models and algorithms only keep approaching this upper limit, so feature engineering is the key to promote the prediction accuracy. Arora & Kaur (2020) use bootstrap-lasso (Bolasso) to select features from a pool of features using consistency and correlation. Jadhav, He & Jenkins (2018) proposed a feature selection algorithm directed by an information gain to select features using GA wrapper (GAW) by ranking features. Pes (2019) applied the ideology of ensemble models to feature engineering by combining feature selection methods, such as filters and embedded methods, univariate and multivariate techniques into a more robust selector. Variance ranking techniques and similarity measures are equally effective methods for feature engineering (Ebenuwa et al., 2019). Priya et al. (2020) used hybrid PCA-GWO for effective feature engineering of DNNs, which improved the convergence speed and reduced the training time. Unfortunately, the feature engineering is a cumbersome affair that relies on a large amount of experimental experience and expert knowledge, which can be disastrous for financial industry practitioners without a data science background. We therefore propose an automatic feature engineering method based on boosting trees and autoencoders.

## Methods and Materials

**Figure 1:**
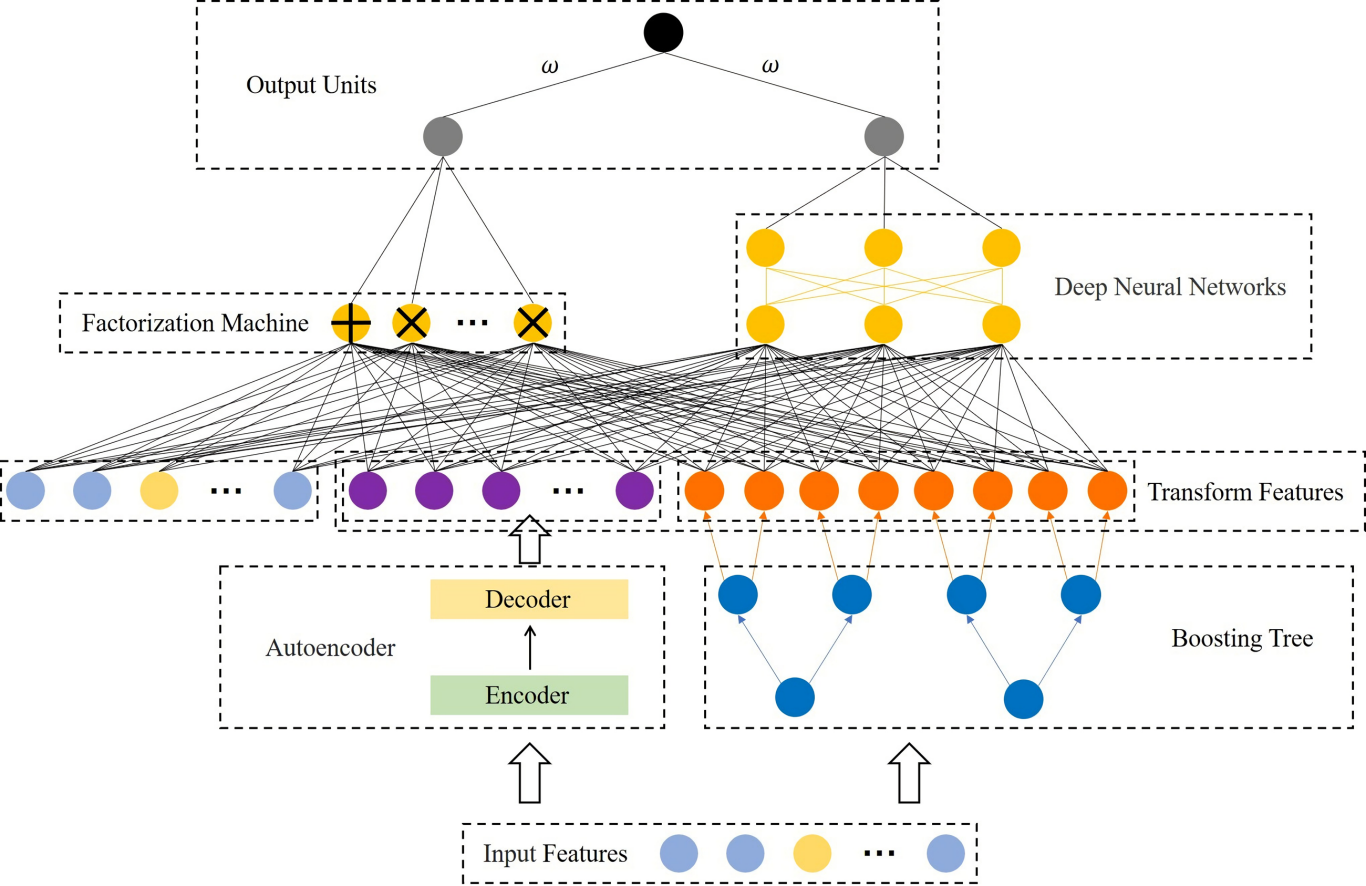
Structural diagram of the proposed model.

In this paper, we propose a cascade model, as shown in Fig. 1. It consists of two parts, the feature transformation model (FTM) and the prediction model (PM). Specifically, the FTM consists of a boosting tree and an autoencoder, the boosting tree is used to generate a higher-order feature representation of the samples to reduce manual feature engineering, while the auto-encoder is used to strengthen the features of a small number of samples to improve the robustness and accuracy in the case of data imbalance. The FTM converts the original sample *x* into $\hat {x}$, as Eq. (1) shows.

(1) \begin{eqnarray*}\hat {x}=x+{f}_{BT} \left( x \right) + \left( x-{f}_{AE} \left( x \right) \right) \end{eqnarray*}

where *f*_*BT*_ and *f*_*AE*_ are the boosting tree module and autoencoder module respectively. Moreover, PM consists of FM and DNN. On the one hand, FM performs feature intersection by matrix decomposition, but is limited by computational complexity and often only conducts second-order feature interaction, so we rely on FM to learn low-order feature interaction. On the other hand, DNN supports a large number of features as input for learning higher-order feature interaction. These two modules are linearly fused to output the prediction results.

### Feature transformation based on boosting tree

Boosting tree is a decision tree algorithm based on boosting, and such kind algorithms include GBDT (Friedman, 2001), XgBoost (Chen & Guestrin, 2016), LightGBM (Ke et al., 2017), CatBoost (Dorogush, Ershov & AJapa, 2018), etc. Decision trees do not rely on normalized feature preprocessing, and the core idea is to go through multiple iterations, with each iteration producing a weak classifier, and each classifier is trained on the residuals of the previous round of classifiers to eventually form a strong learner. The specific implementation of the binary classification GBDT with log-likelihood as the loss function is shown in Algorithm 1.

**Table:** 

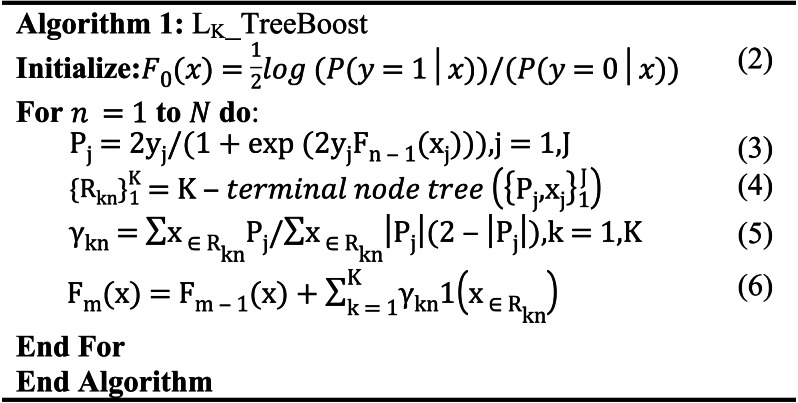

To improve the accuracy of the classifier, there are some tricks to transform the input features of the classifier. For continuous features, Binning is a common method, which bin the feature and treat the bin index as a new categorical feature. Classifier can effectively learn the feature non-linear map. There are various ways of binning data which include fixed-width and adaptive binning. For category features, usually there are two types of categorical variables—nominal and ordinal. There is a certain order between definite ordinal features, and the encoding or mapping scheme can be defined according to the internal order. There are no such connections between adjacent nominal features, and the common approach is to prepare a corresponding value for each category and then perform a unique thermal encoding to eliminate the size difference of the values. However, boost tree feature transformation is a convenient and effective method that enables the transformation of continuous and category features. Boost trees are able to automatically perform feature filtering and combination to generate new discrete feature vectors (Hothorn, 2020). In addition, boosting trees and classifiers are trained independently without joint training, and there is no gradient slew from the classifier to the boosting tree, which reduces the training complexity.

Boosted trees generate multiple subtrees during training, and we treat each tree as a sparse feature with the index of the leaf node where the sample eventually falls into each tree as the value for automatic feature combination to form a new feature vector, which often has a stronger information representation than the original features (He et al., 2014). Suppose the dataset *x*_*i*_ = {*x*_1_, *x*_2_, *x*_3_, …, *x*_*N*_}. The decision tree feature transformation will map *x*_*i*_ to *y*_*i*_ = {*y*_*i*1_, *y*_*i*2_, *y*_*i*3_, …, *y*_*iT*_}, *T* is the number of trees generated by the boosting tree during the training process. *y*_*i*_ is the new feature vector of the original sample *x*_*i*_ after the decision tree feature transformation. *y*_*iT*_ denotes the encoding of the position of the ith sample falling in the *T-th* tree.

**Figure 2:**
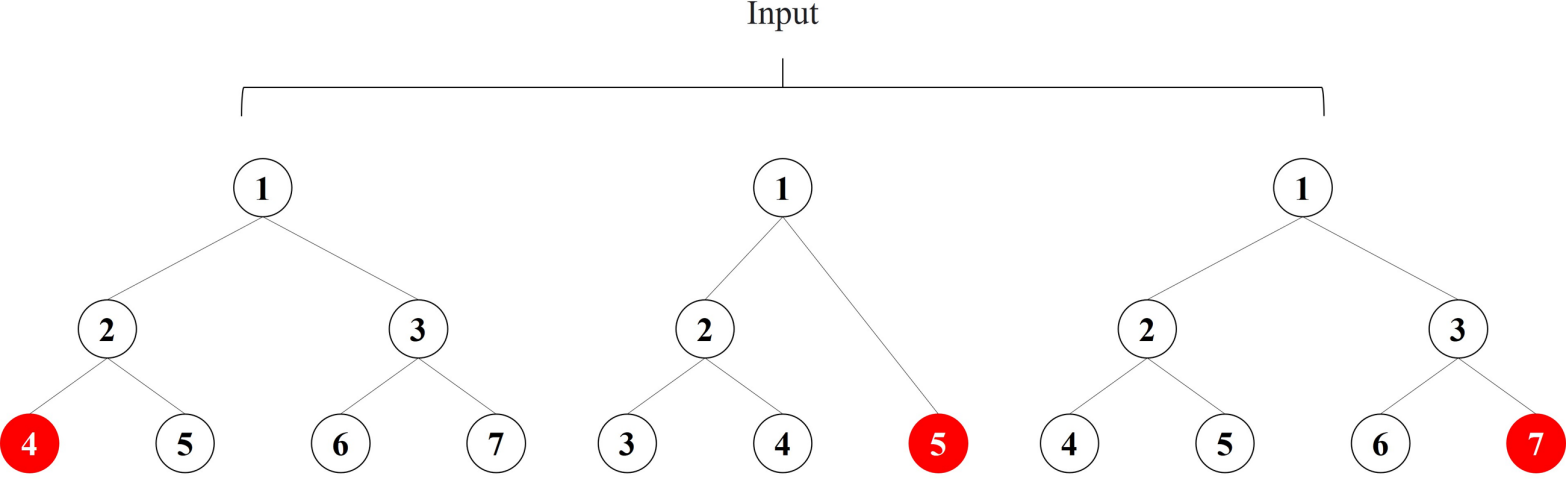
Example of boosting tree feature transformation.

If the boosted tree model generates three subtrees and the sample *x* ends up at node 4 of the first subtree, node 5 of the second subtree, and node 7 of the third subtree, we obtain the new feature vector [4,5,7] of the sample *x*, as shown in Fig. 2. When training a boosted tree model, the number of subtrees is often limited to avoid overfitting, so the discrete feature vector after the decision tree feature transformation does not increase the training difficulty of the model; on the contrary, the effective new features can accelerate the model convergence. The node index of each tree is unique, and we need to recode the new feature vectors, which will generate a large number of high-dimensional sparse vectors after one-hot, which will make the PM difficult to train. Therefore, we add an embedding layer after boosting the number of features transformed to generate low-dimensional dense vectors and accelerate the model convergence. In our experiments, we found that different boosted tree models have similar feature conversion effects, so this paper employs the most widely used Xgboost as the boosted tree feature conversion model.

### Reconstruction error feature algorithm based on autoencoder

Autoencoders are neural networks trained by unsupervised learning, which are trained to learn how to reconstruct data close to its original input (Luo et al., 2019). The autoencoder consists of two parts, namely the encoder and the decoder, and its principle can be described as Eqs. (7)–(9):

(7) \begin{eqnarray*}{f}_{\theta }(x)=\sigma ({W}_{xh}x+{b}_{xh})=h\end{eqnarray*}

(8) \begin{eqnarray*}{g}_{\varphi }(h)=\sigma ({W}_{hx}h+{b}_{hx})=z\end{eqnarray*}

(9) \begin{eqnarray*}E={|}{|}x-z{|}{|}\end{eqnarray*}

where *f*_*θ*_ is the activation function of the encoder, *g*_*φ*_ is the activation function of the decoder, *W* and *b* are the weights and biases of the neural network, while *σ* is the nonlinear conversion function.

The autoencoder maps the input vector *x* into the hidden layer *h* by a nonlinear affine transformation, and the decoder reconstructs the hidden representation *h* toward the original input by the same transformation as the encoder. The difference *E* between the original input *x* and the reconstructed output *z* is referred to as the reconstruction error. The autoencoder continuously optimizes the parameters during the training process to reduce the reconstruction error.

Processing real user credit data is costly due to its high dimensionality and extreme data imbalance (Misra et al., 2020). The usual feature selection and feature extraction methods are computationally expensive to run on large datasets (Ghosh et al., 2018) and statistical filtering based methods ignore the complex connections between multiple features. Therefore, we need a method that can focus on meaningful features from a large number of features and can efficiently learn the feature distribution of unbalanced data. Autoencoder can detect complex nonlinear relationships hidden in data and is not affected by redundant features, so we choose to use autoencoder for feature transformation.

**Figure 3:**
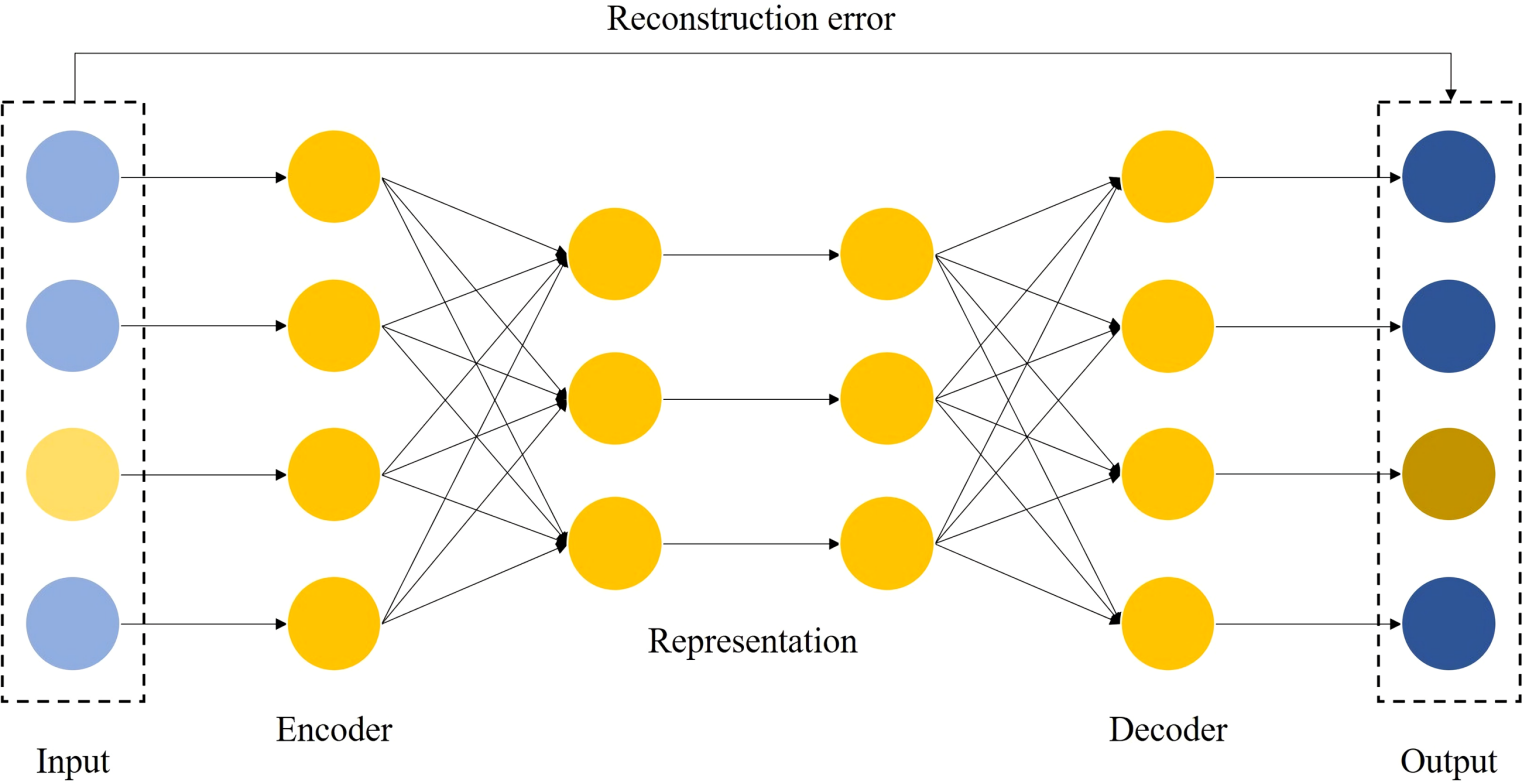
Auto-encoder based feature transformation.

The autoencoder-based feature transformation algorithm is a bias-based approach using semi-supervised learning. It uses reconstruction errors as scores, as shown in Fig. 3. We train the autoencoder using only samples of low credit risk customers. After training, the autoencoder learns primary information about low credit risk customers and can reconstruct them well. In contrast, the autoencoder fails to reconstruct when it encounters a sample of high credit risk customers that it has never seen before. We feed the reconstruction error output from the autoencoder into the prediction model as new features, as implemented in Algorithm 2. In the case of data imbalance, a small number of high-risk customers are reconstructed by the autoencoder and show different feature expressions from those of low-risk customers, which is an effective automatic feature engineering method to make the features of high credit risk customers more significant.

**Table:** 

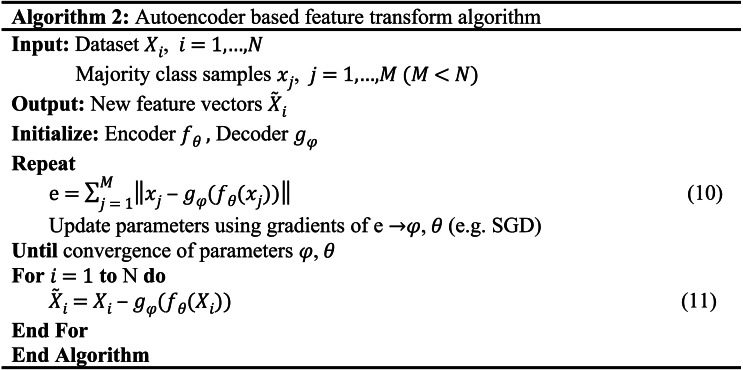

### Factorization machine component

In traditional linear models such as LR, individual features are independent of each other, and if we want employ the classifier to learn the relationship hidden in the features that do not appear in the training set, we need to interact with features artificially, which is a very tedious process; although nonlinear SVM is capable of kernel mapping of features, SVM is not qualified for high-dimensional sparse data. Factorization Machine is a general machine learning model that combines SVM and factorization (Rendle, 2010), which introduces crossover features on the basis of linear model to better mine the association between features and reduce the workload of manual feature interaction. The equation of FM consists of linear units and multiple inner product units, as shown in Eq. (12).

(12) \begin{eqnarray*}y \left( x \right) ={\omega }_{0}+\sum _{i=1}^{n}{\omega }_{i}{x}_{i}+\sum _{i=1}^{n}\sum _{j=i+1}^{n} \left\langle {V}_{i},{V}_{j} \right\rangle {x}_{i}{x}_{j}\end{eqnarray*}

where *n* is the number of sample features, *ω* means the model parameter, ${\omega }_{0}+{\mathop{\sum }\nolimits }_{i=1}^{n}{\omega }_{i}{x}_{i}$ represents the usual expression for linear regression, $ \left\langle {V}_{i}{V}_{j} \right\rangle $ is the dot product, and the latent vector *V* indicates the low-order dense expansion of the feature *x*_*i*_, in fact the length *k* of *V* is usually less than *n*.

In high-dimensional sparse data, there are usually not enough samples to estimate the interrelationships among all features and samples. FM destroys the independence of interaction parameters by factorization, where each interaction does not use its own parameter *ω*_*ij*_, but is modeled by dot product. This allows each feature interaction to help the model to estimate the weights of other feature interactions. FM learns feature interactions that never or rarely appear in the training data very well by training hidden vectors. The binarized features are mapped to a sequential low-dimensional space, and the interaction information between features is obtained by vector inner product. It reduces the complexity of the algorithm while extracting feature interactions and can effectively solve the learning problem of high-dimensional sparse features.

## Results

### Dataset

#### Dataset A: bank loan dataset

The dataset (https://www.kaggle.com/c/GiveMeSomeCredit/overview), derived from user loan information provided by a commercial bank for predicting the likelihood of a user experiencing a financial crisis in the next two years, as shown in Table 2, records various user profile information, such as the gender, age, income, educational background, repayment behavior, overdue behavior and family situation.

#### Dataset B: credit card dataset

**Figure 4:**
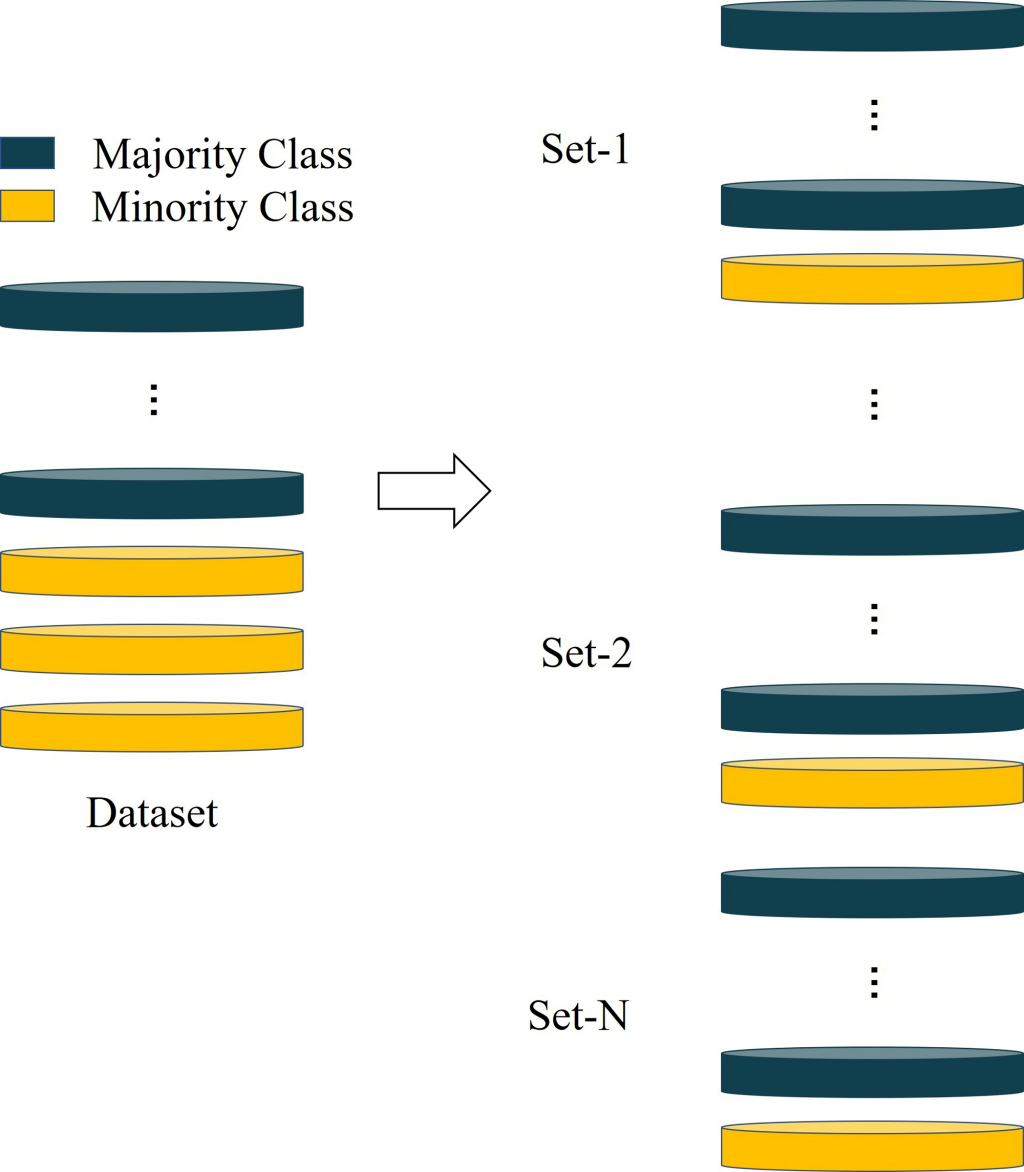
The method for constructing unbalanced dataset.

This data is from the UCI Machine Learning Repository (http://archive.ics.uci.edu/ml). It contains information on default payments, demographic factors, credit information, payment history and bill statements of credit card customers in Taiwan from April to September 2005. Often, an imbalance rate that more than 10 is regarded as extreme imbalance (Jing et al., 2019). Some individuals are sampled from the high credit risk customers and then combined with all low credit risk customers to form multiple imbalance datasets to conduct experiments, as shown in Fig. 4.

**Table 2:** Experiment data.

Dataset	Number of samples	Imbalance rate	Number of features
Dataset A	150,000 (139,974/10,026)	1:13.96	59
Dataset B	30,000(23,364/3,636)	1:6.42	25

### Experiment

The commonly used accuracy (ACC) metric is deceptive and cannot correctly reflect the accuracy of the model. For example, in the case of unbalanced data, where low-risk users account for 99% of the total and high-risk users only account for 1%, if the classifier recognizes all users as low-risk users and then 99%accuracy is achieved. However, it is clear this classifier’s is not qualified. Therefore, in this paper, we use area-under-the-curve (AUC) and logistic loss (Logloss) as the evaluation criteria. AUC represents the area under the ROC graph, which is a method to judge the performance of binary classifiers (Santos, Nedjah & Mourelle, 2018), it does not depend on the threshold setting and is calculated based on the prediction probability. Logloss reflects the average classification bias and is shown in Eq. (13).

(13) \begin{eqnarray*}Logloss=-logP \left( Y{|}X \right) =- \frac{1}{M} \sum _{i=1}^{M}({y}_{i}log{P}_{i}+(1-{y}_{i})\log \nolimits (1-{P}_{i}))\end{eqnarray*}

where *M* is the number of samples, *y*_*i*_ means the true category of sample *x*_*i*_, *P*_*i*_ represents the probability that the classifier recognizes *x*_*i*_ as category 1. If the classifier is an ideal one, the value of Logloss is zero.

We compared the commonly used models in credit default prediction on two datasets, as shown in Table 3. The proposed model achieves AUCs of 0.89 and 0.82 and Loglosses of 0.15 and 0.42 on the two datasets, respectively, significantly outperforming the existing state-of-the-art models. Compared with DeepFM and FNN, which also combine FM and DNN, our model improves the performance by up to 0.18%, which indicates that the proposed method can effectively mine the information of feature interactions. Compared with the sampling techniques of SMOTE and Random Under-Sampling (RUS), our model improves the performance by up to 0.06%, which illustrates the effectiveness of the proposed method in addressing the learning difficulties caused by data imbalance. Compared with models proposed in recent years (Huang, Zhang & Zhang, 2019; Pan et al., 2018; Song et al., 2019; Yang et al., 2020), our model can automatically handle data imbalance and feature engineering with higher accuracy and better applicability.

**Table 3:** Experiment comparison.

	Dataset A		Dataset B	
	AUC	Logloss	AUC	Logloss
SVM	0.62823	0.22808	0.73049	0.45938
GBDT	0.83224	0.18776	0.77713	0.43253
LR	0.79268	0.22551	0.72056	0.46841
XGB	0.86443	0.18337	0.78052	0.52310
GNB	0.79449	0.49821	0.73850	1.01296
RF	0.83786	0.19450	0.75198	0.48226
DNN	0.83012	0.18844	0.76903	0.44012
FM	0.79245	0.20463	0.74665	0 .56924
XGB+LR	0.84422	0.19262	0.75486	0.43509
SMOTE+XGB	0.88312	0.19465	0.79413	0.40965
RUS+XGB	0.85471	0.26135	0.77458	0.44085
DeepFM	0.82884	0.18840	0.77562	0.43401
FNN	0.82847	0.18856	0.77271	0.43887
DCN	0.82749	0.18928	0.77463	0.43493
AutoInt	0.82798	0.18853	0.77514	0.43417
FwFM	0.82867	0.18861	0.77515	0.43893
FiBiNET	0.82629	0.18964	0.77604	0.4351
ONN	0.82802	0.19042	0.75828	0.45015
**OURS**	**0.89794**	**0.15223**	**0.82736**	**0.42237**

**Figure 5:**
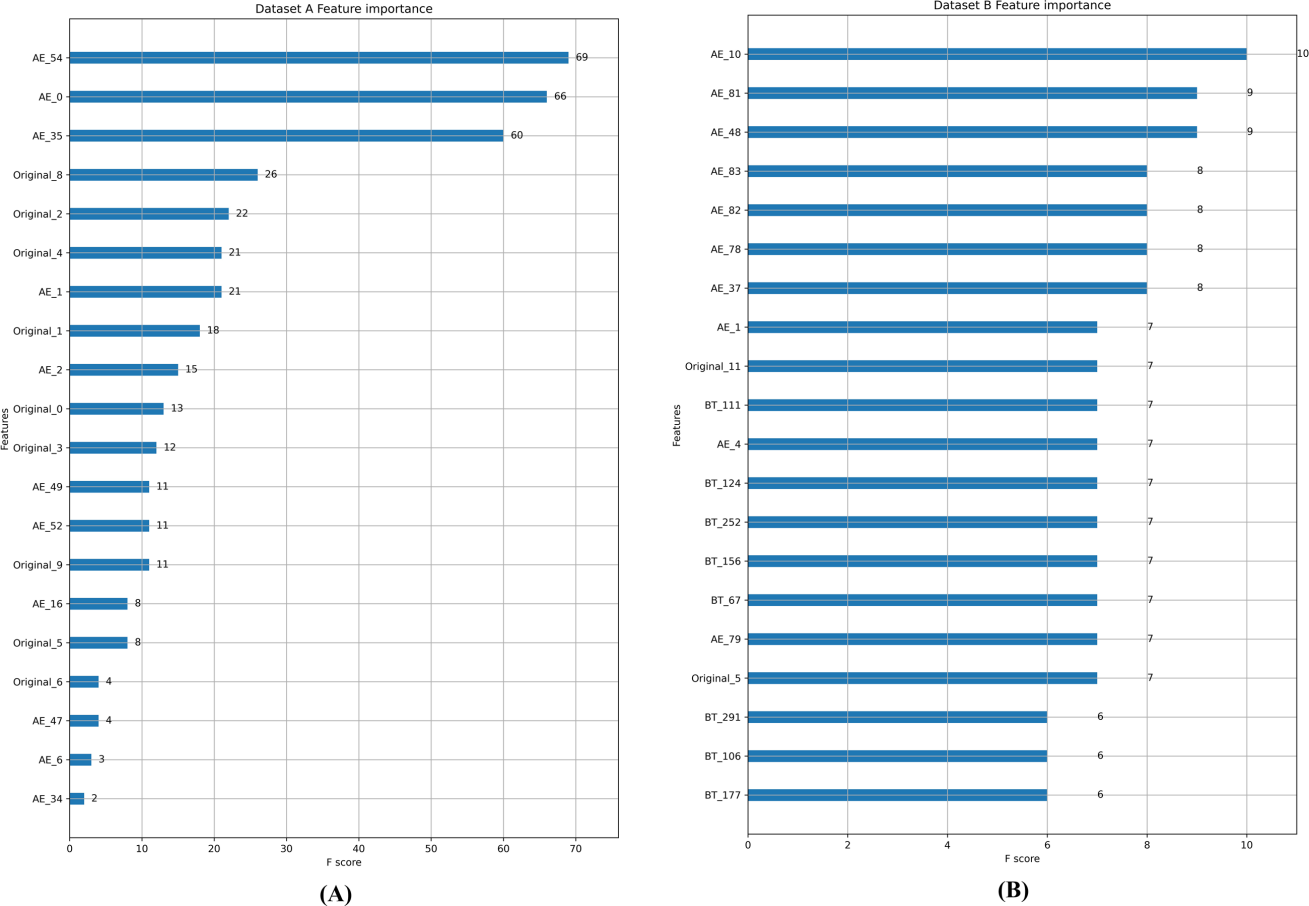
(A-B) Feature importance comparison.

Feature importance ranking in random forest is conducted, as shown in Fig. 5, and it can be observed that most of the top 20 features are generated by FTM. In Dataset A, the feature weights of the reconstructed error features generated by the autoencoder account for about half of the entire feature set. In Dataset B, only two of the top 20 features are from the original feature set. This verifies that the feature enhancement and feature transformation of FTM is effective in the case of extremely imbalanced data.

## Discussion

In the actual credit scoring, the laborious feature engineering and data imbalance are the urgent issues should be overcome. Due to the dimensionality and sparsity of data provided by different organizations and different businesses, existing models are not qualified. The proposed method can effectively solve these problems. We first use boosting trees and autoencoders for features to modify the relative distribution of data and enhance the differentiation between different categories. Regardless of the feature representation of the original data, it can be transformed into a discrete representation of the boosted tree and a continuous representation of the autoencoder by FTM, which reduces the impact of different dimensions of the original features on the prediction results and improves the robustness of the model. It can effectively transform the higher-order feature expressions and enhance the discrimination between different categories of samples. In our proposed integrated model, FM and DNN share feature-transformed inputs and perform parallel training followed by weighted fusion to output prediction results, which enables sufficient low-level feature interaction and high-order feature interaction. As can be seen in Table 3, the proposed method is able to significantly improve the precision of prediction compared with other methods and only requires the input of raw features. This is of great interest for realistic work.

## Conclusion

In this paper, we propose an ensemble model based on the scheme of feature transformation combining FM and DNN for credit scoring, finally achieving the state-of-the-art performance. The proposed approach performs automatic feature engineering through boosting trees and autoencoders, and then learns both low-order feature interactions and high-order feature interactions through parallel training of FM and DNN. Comparison experiments were conducted on two real datasets, and the results indicate that the proposed model can not only effectively solve the difficulties of data imbalance and feature engineering, but also provide reliable performance in credit default prediction. In the future, on the one hand, we plan to release a standard dataset for peers’ study, on the other hand, an advanced GPU cluster is aimed to build to explore the large-scale data prediction task.

## Additional Information and Declarations

### Data Availability

The following information was supplied regarding data availability: Dataset A is available at Kaggle: https://www.kaggle.com/c/GiveMeSomeCredit/overview, “Give Me Some Credit”. Kaggle, 2021, doi: 10.34740/KAGGLE/DSV/2242482. Dataset B is available at the UCI Machine Learning Repository:
https://archive.ics.uci.edu/ml/datasets/default+of+credit+card+clients. Code is available at GitHub: https://github.com/warden-L/A-Feature-Transformation-and-Ensemble-Model-Based-Approach-for-Credit-Scoring.
